# The Anticancer Efficacy of Thiourea-Mediated Reduced Graphene Oxide Nanosheets against Human Colon Cancer Cells (HT-29)

**DOI:** 10.3390/jfb13030130

**Published:** 2022-08-27

**Authors:** Babu Vimalanathan, J. Judith Vijaya, B. Carmel Jeeva Mary, Savarimuthu Ignacimuthu, Magesh Daniel, Ramasamy Jayavel, Mohamed Bououdina, Stefano Bellucci

**Affiliations:** 1 Crystal Growth Centre, Anna University, Chennai 600025, India; 2 Catalysis and Nanomaterials Research Laboratory, Department of Chemistry, Loyola College, Chennai 600034, India; 3 Xavier Research Foundation, St. Xavier’s College, Palayamkottai, Thirunelveli 627002, India; 4 Department of Zoology, Loyola College, Chennai 600034, India; 5 Department of Mathematics and Sciences, Faculty of Humanities and Sciences, Prince Sultan University, Riyadh 122001, Saudi Arabia; 6 INFN-Laboratori Nazionali di Frascati, Via E. Fermi 54, 00044 Frascati, Italy

**Keywords:** thiourea, reduced graphene oxide, flow cytometry analysis, colon cancer cells, DNA fragmentation, scanning electron microscopy

## Abstract

The current research focuses on the fabrication of water-soluble, reduced graphene oxide (rGO) employing thiourea (T) using a simple cost-effective method, and subsequently examining its anticancer characteristics. The cytotoxicity caused by graphene oxide (GO) and T-rGO is investigated in detail. Biological results reveal a concentration-dependent toxicity of GO and T-rGO in human colon cancer cells HT-29. A decrease in cell viability alongside DNA fragmentation is observed. Flow cytometry analysis confirms the cytotoxic effects. The novelty in this work is the use of raw graphite powder, and oxidants such as KMNO_4_, NaNO_3_, and 98 percent H_2_SO_4_ to produce graphene oxide by a modified Hummers method. This study demonstrates a simple and affordable procedure for utilising thiourea to fabricate a water-soluble reduced graphene oxide, which will be useful in a variety of biomedical applications.

## 1. Introduction

Graphene with *sp*^2^-bonded carbon atoms in a planar sheet with a honeycomb crystal lattice is attracting considerable interest because of its potential applications due to the unexpected approach of connecting condensed matter and quantum field theory [1]. The physical, chemical, electrical, optical, and mechanical features of graphene are outstanding [2,3,4], in addition to being biocompatible [5,6]. Transistors of thin sheets, touch panes, and stellar cells [2,3,4,7], nanoelectronics [8,9], sensors [10,11], nanocomposites [12,13], batteries [14], supercapacitors, hydrogen storage [15], anticancer drugs [16], drug delivery [17], tissue engineering [18], antibacterial composites [19], bio-sensing [20], and other technological sectors might benefit from this unique nanostructure. Alternatively, there exists a variety of reactive oxygen functional groups, which makes graphene-based materials useful in bioengineering [21]. Among graphene derivatives, graphene oxide (GO) offers additional features such as minimal manufacturing costs, a high surface, excellent viscous behavior, and reduced cytotoxicity. The GO liquefaction in solvents, particularly water, is critical for bioengineering applications. In solvent, the maximal solubility of GO is achieved by the polarity of the solvent, as well as the extent of surface functionalization given during the oxidation process.

There are several approaches for synthesizing GO; a modified Hummers process is the most prevalent chemical route [22]. The GO surface produced by the above-mentioned Hummers process contains oxygen functional groups such as carboxyl, epoxy, and hydroxyl, allowing it to disperse easily in water [23]. A considerable number of studies demonstrate that GO materials, such as GO films (paper), function as excellent biocompatible materials, which allow human and animal cells to proliferate well, with little or no cytotoxicity. Such properties appear to imply that GO materials might be utilized in tissue implants, tissue engineering, and wound treatment applications. Multiple research groups were inspired by these unique characteristics to better define the cytotoxic and antimicrobial capabilities of GO. Numerous recent investigations found that GO paper improves the engraftment of L-929 cells [24], osteoblasts [25], kidney cells, and embryonic cells [26]. A variety of substances, including amino acids [27], glucose [28], and melatonin [29], were found to decrease the efficacy of GO. In the present study, using thiourea as a reductive agent, a novel method for the simultaneous reduction and functionalization of GO was developed. This is a straightforward and cost-effective approach. Thiourea, as an organosulfur chemical, was chosen because it contains amine groups that are important for removing the oxygen functional group from graphene oxide.

Hence, thiourea was chosen for GO reduction. Carbon-based nanomaterials, such as pure graphene and its derivatives, demonstrate potential application in the medical field; hence, it is critical to analyze their toxic potential in mammals’ cells [30,31].

Akhavan et al. [32] studied the toxicity of reduced graphene and GO oxide nanowalls against the bacterium *Staphylococcus aureus*. Both graphene derivatives prove their effectiveness as antibacterial constituents due to the direct contact between the bacteria’s cell wall membrane and the very sharp edges of the graphene sheets. Graphene or GO sheets were tested for toxicity against various cell types, including cancer cells. In cancer cells of the human breast [33] and cancer cells of the ovary [34], our research team found that physiologically, a lower amount of graphene causes more toxicity. Zhang et al. [35] used a combination of photothermal and chemotherapy agents to demonstrate the anticancer impact of NGO–PEG–DOX (nanoscale graphene oxide–polyethylene glycol–doxorubicin). A chemo–photo–thermal therapy combination has a synergistic impact, resulting in a greater influence of killing the cancer cells than either mere light thermal therapy or chemotherapeutic measures. 

Cancer is the leading cause of death in developed countries, especially among the poor population [36]. Colon cancer is the third most common malignancy and the fourth largest cause of death worldwide, as per the International Agency of WHO (world health organization) for Research on Cancer [37]. It grew into a commonly prevalent form of cancer worldwide [38]. It is the most diagnosed cancer worldwide [38], with 140,000 fresh cases and 50,830 mortalities in the United States in 2013, and more than 1.2 million fresh cases and 600,000 casualties globally [39]. Chemotherapy is the most prevalently used therapeutic technique in treating cancer; however, when administered alone, it does not provide a good therapeutic outcome. The most aggressive tumor, with significant morbidity and death rates, and a dismal prognosis [40], is colon cancer. Colon cancer is seen as a condition that may be avoided [41]. However, there appears to be no decrease in the incidence of colon cancer, and many of the risk factors for colon cancer continue to exist. Colon cancer is treated with surgery, radiation, and chemotherapy [42]. However, due to resistance to chemotherapy, toxicity, and side effects, such therapies are insufficient. As a result, it is critical to develop innovative anticancer medicines with fewer side effects to meet patients’ unmet treatment needs. Lack of selectivity, systemic toxicity, and chemoresistance are the limitations of conventional chemotherapy approaches to cancer. Nanomaterials such as graphene could offer more targeted cancer treatment, successfully decreasing the undesirable side effects, and offering accurate diagnosis and effective therapy.

To find out the anticancer potential of thiourea-reduced graphene oxide (T-rGO) for the treatment of colon cancer, the challenge was to design cost-effective and reactive lead molecules with enhanced responsiveness and target cell selectivity. The aim of this research consists of developing a simple, reproducible, and cost-effective method for reducing and functionalizing GO utilizing thiourea, and assessing their toxicity in colon cancer cell line HT-29 by examining cell survival, DNA fragmentation, and by flowcytometry. The findings indicate that thiourea-mediated GO reduction exhibits a low toxic effect and high cell death activity against the cell line of colon cancer. Reduced graphene oxide mediated by organic molecules may pave the way for new routes to employ thiourea for graphene synthesis, with the byproduct potentially being used for anticancer treatments.

## 2. Methodology

Graphite powder, NaNO_3_ anhydrous ethanol, NaOH, KMnO_4_, 36% HCl, 98% H_2_SO_4_, 30% hydrogen peroxide (H_2_O_2_), and aqueous solution of graded quality were purchased from Sigma-Aldrich (Chennai, India), and utilized without additional purification. HT-29 human colon cancer cell line was obtained from NCCS, Pune; MEM medium and antibiotics were procured from Sigma-Aldrich, India; and 10% FBS (heat-inactivated fetal bovine serum) was secured from National centre for cell science (Pune, India). De-ionized water was used to make all aqueous solutions. Unless otherwise specified, all additional compounds were acquired from Sigma-Aldrich.

### 2.1. Synthesis of GO

With some changes, the previously published method (22) was used to synthesis GO (70). It was an ordinary synthesis procedure. A total of 350 mL of chilled (0 °C) H_2_SO_4_ was combined with 2 g of natural Gt powder, 8 g of KMnO_4_, and 1 g of NaNO_3,_ while being stirred. The mixture was put into a water bath at 40 degrees and agitated for 60 min. While slowly adding 250 mL of deionized water, the temperature was raised to 98 °C. After 30 min of keeping the mixture at 98 °C, the reaction was stopped by adding 40 mL of 30% H_2_O_2_ and 500 mL of deionized water. The mixture’s color turned to a dazzling yellow, showing that pristine Gt was converted to GO. To remove metal ions, the mixture was filtered and rinsed with diluted HCl. After achieving a pH of 7.0, the product was repeatedly rinsed with distilled water, and the synthesized GO was then sonicated for 60 min. The brown GO sheet’s subsequent aqueous dispersion was dependable.

### 2.2. Synthesis of rGO

A total of 500 mg of GO was dispersed in 250 mL of water using ultrasonication for 1 h and 30 m in a typical reduction experiment, yielding GO dispersion. Under constant stirring, 500 mg of thiourea was added to the dispersion, which was then refluxed at 94 °C for 24 h. The brown hue of GO dispersion turned black throughout the reduction phase.

### 2.3. Characterization of rGO and T-rGO

As previously stated in the literature [34,43,44,45], in-depth characterization was carried out. A3 kW X-ray diffractometer Rigaku Miniflex II-C (Rigaku, Singapore) was used to record X-ray diffraction (XRD) patterns in the region of 2θ = 5–80°, using a scintillation counter, Cu target (λ = 1.5418 Å), and operating at 40 kV and 40 mA. TESCAN Vega 3 (TESCAN, Bron, Czech Republic) scanning electron microscope (SEM) was used to characterize particle morphology and size. The powdered samples were fixed onto carbon tape and subsequently coated with gold layer for better imaging. The Agiltron Peak Peaker Pro 532 (Agiltron, Woburn, MA, USA) was used to analyze the Raman spectra of GO and T-rGO using a 532 nm laser. The calibration was performed at 500 cm^−1^ with an inbuilt silicon standard, resulting in a less than 1 cm^−1^ of peak position resolution. The Raman spectra were measured in the range of 500−4500 cm^−1^. Without using any solvent, all materials were powdered and placed on glass slides. FTIR spectroscopy was used to identify the functional groups by means of Jasco FTIR 6600. The sample’s thermal stability was studied using EXSTAR S11 TG/DTA 6300 (Seiko instruments Inc., chiba, Japan) thermogravimetric analysis up to 1000 °C with a heating rate of 10 °C/min under nitrogen environment.

### 2.4. Cell Line and Culture

NCCS, Pune, provided the HT-29 cell line. The cells were maintained in a humidified environment of 50 g/mL CO_2_ at 37 °C in minimal essential medium that was supplemented with penicillin (100 U/mL), 10% FBS, and streptomycin (100 g/mL) [46]. 

### 2.5. Reagents

Minimum Essential Medium, fetal bovine serum (FBS), and dimethyl sulfoxide (DMSO) were obtained from Hi-Media Laboratories (Mumbai, India) Methylthiazolyldiphenyl-tetrazolium bromide (MTT), DMSO, and trypsin were purchased from Cistron Lab (Sisco Research Lab Chemicals, Mumbai, India). Sigma-Aldrich Mumbai, India provided all the other chemicals and reagents.

### 2.6. WST-8-Cell Viability Assay

The WST-8 test was used to determine cell viability, as previously described [33,47]. In a 96-well plate, 1 × 10^4^ cells were planted in 100 µL of 10% FBS-containing MEM. After being washed two times with serum-free MEM of 100 L, the cells were cultured for 24 h in 100 mL of serum-free MEM, with various concentrations of GO or T-rGO solutions ranging from 1000 to 7.8 g/mL. The cells were washed twice with serum-free MEM after 24 h of exposure, and 15 µL of the WST-8 solution was added to each well containing 100 µL of serum-free MEM. Since a reduction in GO or residual GO could impact the measured absorbance at 450 nm, with the aid of microplate reader, 80 µL of mixture was moved to another 96-well plate after 1 h of incubation. To see if GO and T-rGO interacted directly with the WST-8 reagent, cell-free control tests were carried out. 

A total of 100 L of GO or T-rGO suspensions in varied quantities (20–100 g/mL) were placed in a 96-well plate, and to every well 10 mL of WST-8 reagent solution was added; the incubation of the mixed solution was performed for 1 h at 37 °C under 5% CO_2_. After incubation, the GO or T-rGO was subjected to centrifugation, and 50 L of the effluent was transferred to a new 96-well plate. The observation of the optical density was performed at 450 nm.

### 2.7. Cell Cycle Arrest Analysis

In a six-well plate, HT-29 cells were plated with 2.0 × 10^5^ cells/mL (density). After an incubation period of 24 h (37 °C, 5% CO_2_), the cells were treated with IC_50_ concentrations of GO (15.6 µg/mL) and T-rGO (31.2 µg/mL) in serum-free medium and incubated for another 24 h. In the cell cycle assay, the IC_50_ derived from the cytotoxicity assay for each treatment was employed. The cell cycle phase analysis was carried out according to Grassi et al. [48]. The cells were trypsinized and centrifuged at 1000× *g* for 10 min before being re-suspended in PBS and collected. The cells were given 0.2 mL of propidium iodide (10 µg/mL) and incubated for 30 min. The cells were examined using flow cytometer at 488 nm (excitation) and at 488 nm (emission) immediately after 30 m of incubation at 37 °C to identify the cell cycle stage. The results provided here are representative of at least three separate tests carried out in triplicate.

### 2.8. DNA Fragmentation

To achieve confluence, plating of the HT-29 cells was carried out in 6 well plates and incubated in a CO_2_ incubator. After collecting the cells with TPVG, 1.5 mL of cell suspension was transferred into an Eppendorf tube. The cells were then centrifuged for 10 min at 200 rpm and at 4 °C. After that, 0.5 mL TTE solution was added to the pellet and vortexed vigorously. The cell lyses (due to Triton X-100 in TTE solution) and breakdown of nuclear structure (due to Mg^2+^ chelation by EDTA in the TTE solution) permits the fragmented chromatin to be released from the nuclei. 

The fragmented DNA was isolated from chromatin by centrifuging at 20,000 rpm for 10 min at 4 °C. After that, the supernatants were removed, and the pellet was washed with 500 µL of TTE solution. Then, 500 µL of ice-cold NaCl was added. Histones were then removed from DNA with the addition of salt. A total of 700 µL ice-cold isopropanol was added and vigorously stirred. The precipitation started after 24 h and the obtained DNA was fabricated in the form of a pellet at 20,000 rpm and at 4 °C. After precipitation, the pellets were rinsed with 500–700 µL ice-cold 70% ethanol. Later, DNA was dissolved by adding TE solution of 20–50 µL to each tube and stored at 4 °C. The DNA samples were loaded with buffer at a concentration of 10×loading buffer to a final concentration of 1 mL/1X.

After adjusting the voltage to the correct level, the samples were loaded into wells more readily with the addition of loading buffer. The samples could be also monitored as they ran through the electrophoresis in conventional TE buffer. The migration of the bromophenol blue dye present in the loading dye was used to track the migration of materials during electrophoresis. The electrophoresis was stopped when the dye reached about 3 cm from the end of the gel. The DNA was observed using UV Trans illuminator [49,50].

### 2.9. Cell Morphology

In 10% FBS-containing MEM, HT-29 plating of cells was conducted using 6-well plates (1 × 10^5^ cells per well). The medium was replaced with serum-free MEM with and without 1000, 15.6, and 7.8 µg/mL of GO for the next 24 h (IC_50_ concentration) and T-rGO concentrations of 1000, 31.2, and 7.8 µg/mL (IC_50_ concentration). The cell morphology was examined by optical microscope (Guilin FT-OPTO Co., Ltd., Guilin, China).

## 3. Results and Discussion

### 3.1. XRD Analysis of GO and T-rGO

**Figure 1:**
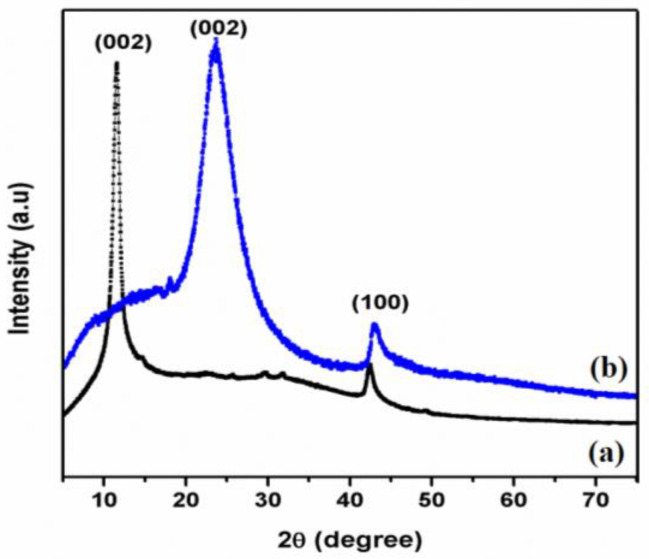
XRD pattern of (a) GO and (b) thiourea-reduced graphene oxide.

XRD can be used to inspect the interlayer alterations and crystalline characteristics of the synthesized material. Figure 1a,b displays the XRD patterns of GO and T-rGO. The d-spacing interlayer is a crucial element in determining the structural information of graphene when it is produced. The strong peaks observed in Figure 1a emerging at 2θ = 11.6° and 42.5° correspond to the reflections (002) and (100) of the typical hexagonal lattice structure of two-dimensional GO. For T-rGO, the same (002) and (100) reflections become much broader and shift toward higher 2θ angles, i.e., 25.8° and 43.4°. This indicates a contraction of the lattice and small crystallite size. The existence of functional groups comprising oxygen formed during oxidation is established by the XRD pattern of GO. The strong peaks in the XRD pattern of T-rGO further indicate that the transition of graphene oxide to rGO is complete [51,52,53,54]. The absence of additional peaks in both GO and T-rGO XRD patterns confirm that both samples are pure and free of contaminants.

### 3.2. Morphological Observations of GO and T-rGO

**Figure 2:**
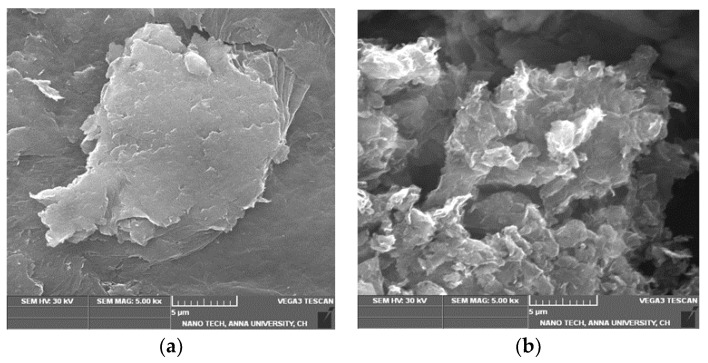
SEM images of (**a**) GO and (**b**) T-rGO.

The surface morphology of graphene derivatives was investigated using scanning electron microscope. The modified Hummers technique was used to construct GO sheets from natural graphite flakes [22]. Both GO and T-rGO are water soluble due to the presence of functional groups having oxygen-containing sheets [55]. Graphite appears to be heaped up in thick powder, whereas GO is filled with peels of thin large flakes with wavy creases [55]. Figure 2a reveals GO sheets with smooth surfaces and minor wrinkles at the borders displaying undulating nature. The SEM images of GO resemble rippling silk waves that are translucent. The oxidation process crumples the edges of the exfoliated GO sheets, whereas the shape of T-rGO is crumpled and paper-like, with a sheet-like structure (Figure 2b). A substantial alteration is noticed at the sharp edges because of enhanced oxidation. The fact that the folded stacking GO structure differs from the bent structure of abridged GO suggests the reduction process of thiourea as an important mechanism in the transition of GO to graphene [56,57,58,59].

### 3.3. FTIR Analysis of GO and T-rGO

**Figure 3:**
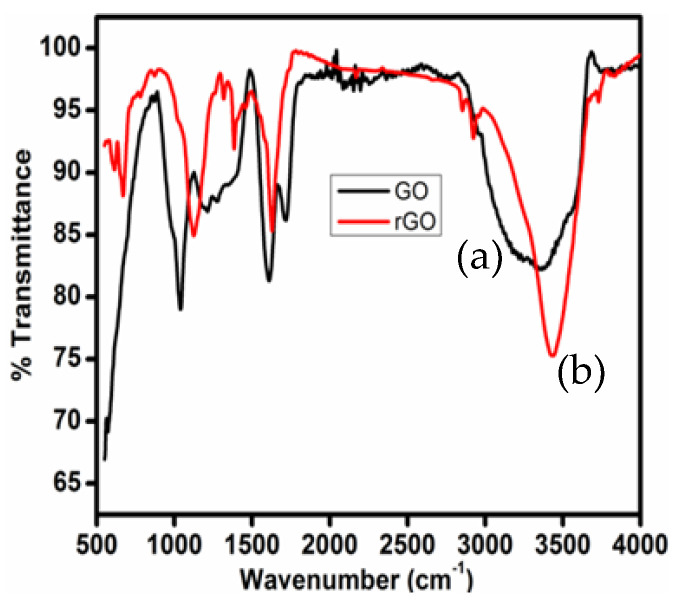
FTIR spectra of (a) GO and (b) thiourea-reduced graphene oxide.

The FTIR technique is useful for confirming the degree of GO reduction. FTIR spectroscopy verifies the decrease in oxygen-containing functional groups in GO and T-rGO. Figure 3 displays the FTIR spectra of GO and T-rGO.

Figure 3a shows the FTIR spectrum of GO; a broad band is observed at 3363 cm^−1^, assigned to the stretching vibrations of the hydroxyl groups. Carbazole groups are deformed with the appearance of a band at 1727 cm^−1^. Unoxidized graphitic domains are identified by skeletal vibrations around 1034 cm^−1^ [60]. The bands located at 1612 and 1034 cm^−1^ are ascribed to epoxy groups (C–O–H deformation–OH stretching) and C–O stretching vibrations (alkoxy groups). An increase in the frequency at 1378 cm^−1^ could be attributed to rGO sheet skeletal vibrations, as reported earlier [61]. The band at 1128 cm^−1^ indicates the existence of some functional groups, such as C-O, C=O, and N-H stretching vibrations; it is important to note that thiourea decreases most of the functional groups, including oxygen. 

### 3.4. Raman Analysis of GO and T-rGO

**Figure 4:**
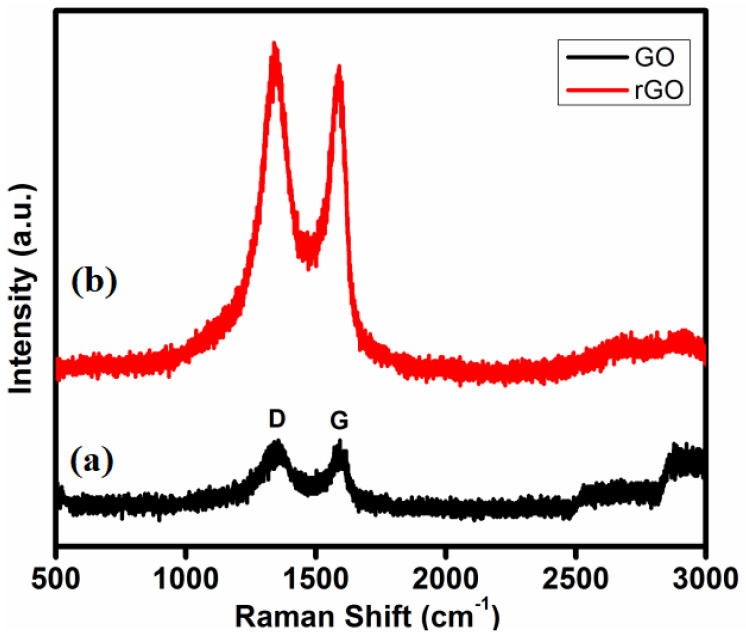
Raman spectroscopy analyses of (a) GO and (b) rGO samples.

Raman spectroscopy is a powerful tool used to confirm and identify the active modes of carbonaceous materials, as well as to discriminate between ordered and disordered structures of carbon crystal [62]. Furthermore, it is proven to be very effective for identifying major structural changes occurring throughout the reduction process from GO to rGO. Figure 4a,b show both GO and T-rGO Raman spectra. For GO, as shown in Figure 4a, the D-band peak at 1356 cm^−1^ is due to the presence of A_1g_ symmetry breathing mode of k-point photons, whereas the G-band peak at 1578 cm^−1^ is due to the first-order scattering of E_2g_ symmetry phonons from sp^2^ carbon atoms, which matches perfectly with the literature.

For T-rGO, as shown in Figure 4b, both the D and G bands have slightly shifted toward lower wave number, i.e., 1350 and 1376 cm^−1^, respectively. When compared to GO, the intensity ratio (I_D_/I_G_) for rGO is found to be greater than that of T-rGO, 1.09 and 0.92, respectively.

Upon post-GO chemical reduction, the conjugation of the sp^2^ carbon network is detected and re-established. Due to a rise in the D/G intensity ratio, the re-established sp^2^carbon has a network size that is generally smaller than the initial graphite layer. The maxima of the other overtone bands, such as the 2D and D+G bands, are observed at 2610 and 2890 cm^−1^, respectively. Four distinct types of layers of rGO sheets are usually identified from Raman spectroscopy analysis, as reported in the literature [62,63]. Notably, the relative intensity changes of the G band to D band in the Raman spectra are detected throughout the reduction of GO, revealing a shift in the electronic conjugation state, and confirming a rise in the number of sp^2^ domains [64,65,66,67].

### 3.5. Thermal Analysis of GO and T-rGO

**Figure 5:**
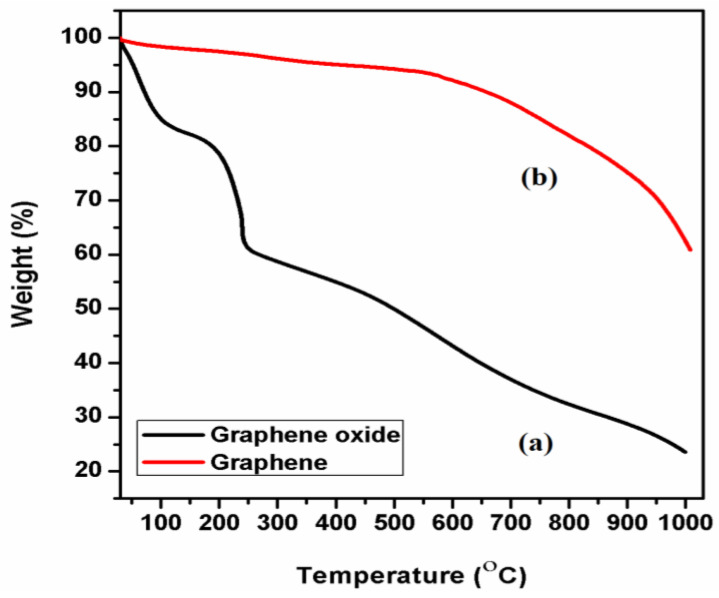
Thermo gravimetric investigation of (a) GO and (b) T-reduced graphene oxide.

The TGA analysis was used to examine the thermal stability of graphene derivatives. Figure 5a,b illustrates TGA curves of GO and T-rGO representing the mass loss during heating under air. 

The elimination of oxygen-containing functional groups such as H_2_O and O_2_ causes a mass loss between 150 and 200 °C at the first stage [68]. Furthermore, the bulk pyrolysis of the carbon skeleton results in further mass loss at temperatures above 300 °C [69]. At temperatures over 600 °C, this is also reported in rGO [70]. T-rGO, on the other hand, has considerably higher thermal stability than pure GO, i.e., with a maximum mass loss percentage (38%) at high temperatures (1000 °C) compared to pure GO sheet (78%). From the present study, it is apparent that the oxygen functional groups are removed from GO sheets’ surface during the reduction process.

### 3.6. Impact of GO on the Potentiality of HT-29 Cells

**Figure 6:**
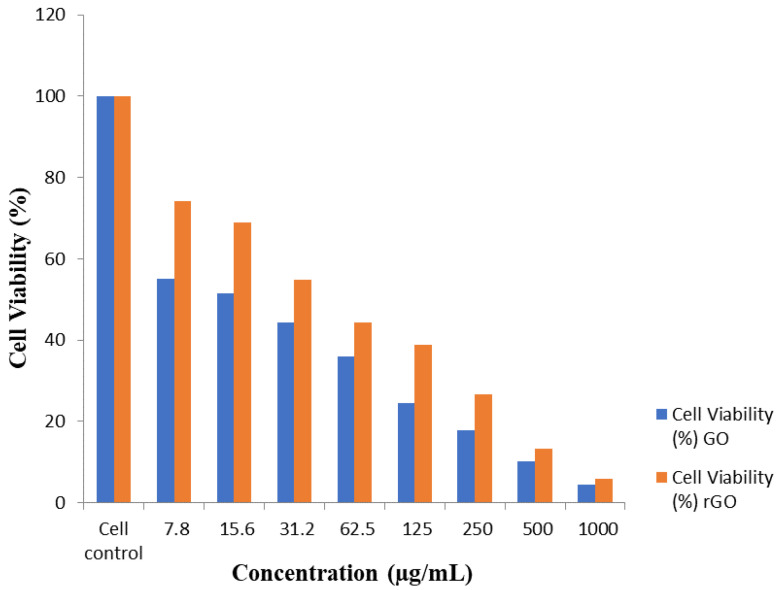
GO and T-rGO effects on cell capability of HT-29 human colon cancer cells. The viability of HT-29 human colon cancer cells was determined after 24 h exposure to different concentrations of GO and T-rGO by means of the WST-8 assay.

The cytotoxicity of GO and T-rGO was examined on HT-29 cells. The effect of GO and T-rGO on cell viability of HT-29 cancer cells, which were cultured for 24 h with various doses of GO and T-rGO ranging from 1000 to 7.8 g/mL, was studied. The viability of HT-29 cells is found to be lowered by both GO and T-rGO, depending on the dose, as illustrated in Figure 6. GO, on the other hand, substantially reduces cell viability as compared to T-rGO. Both graphene derivatives manifest anticancer activity in colon cancer cells, but GO has an effect of 51.60% at the concentration of 15.6 µg/mL (IC_50_ concentration of GO) compared to T-rGO with 54.80% when the concentration is 31.2 µg/mL (IC_50_ concentration of T-rGO) (Table 1 and Table 2). The obtained results show that GO is cytotoxic at concentrations as low as 15.6 µg/mL. Furthermore, these results are better than S. Gurunathan et al. (2016), where cytotoxicity was found to occur at a minimum concentration of 20 µg/mL [70].

Pure graphene causes cytotoxicity by lowering the potential of the mitochondrial membrane, boosting intracellular ROS production, and then activating the mitochondrial pathway as a consequence, causing apoptosis. The results were calculated using the average standard deviation of three separate studies [71]. The vitality of T-rGO and GO treated cells varies considerably from that of untreated cells, as verified in student’s *t*-test (*p* < 0.05).

### 3.7. Graphene-Induced Arrest at the Sub-G_1_ and G_1_ Phases of the Cell Cycle

**Figure 7:**
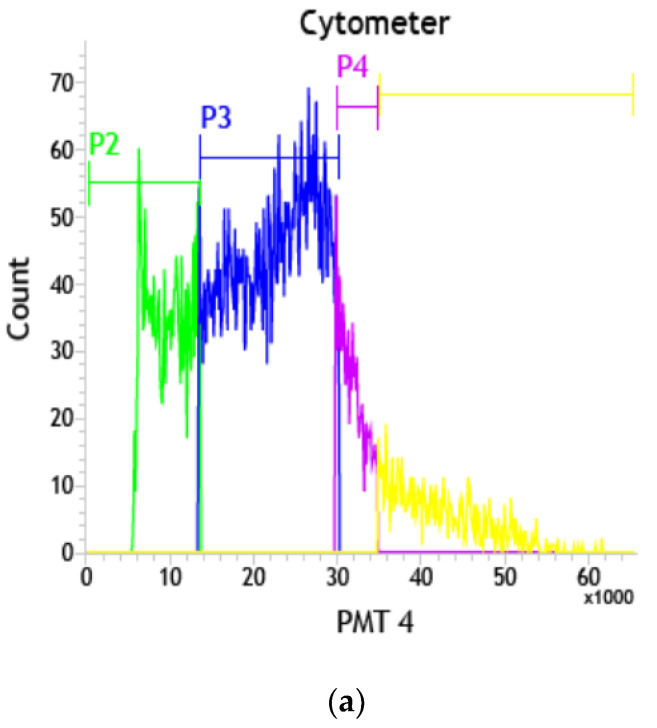

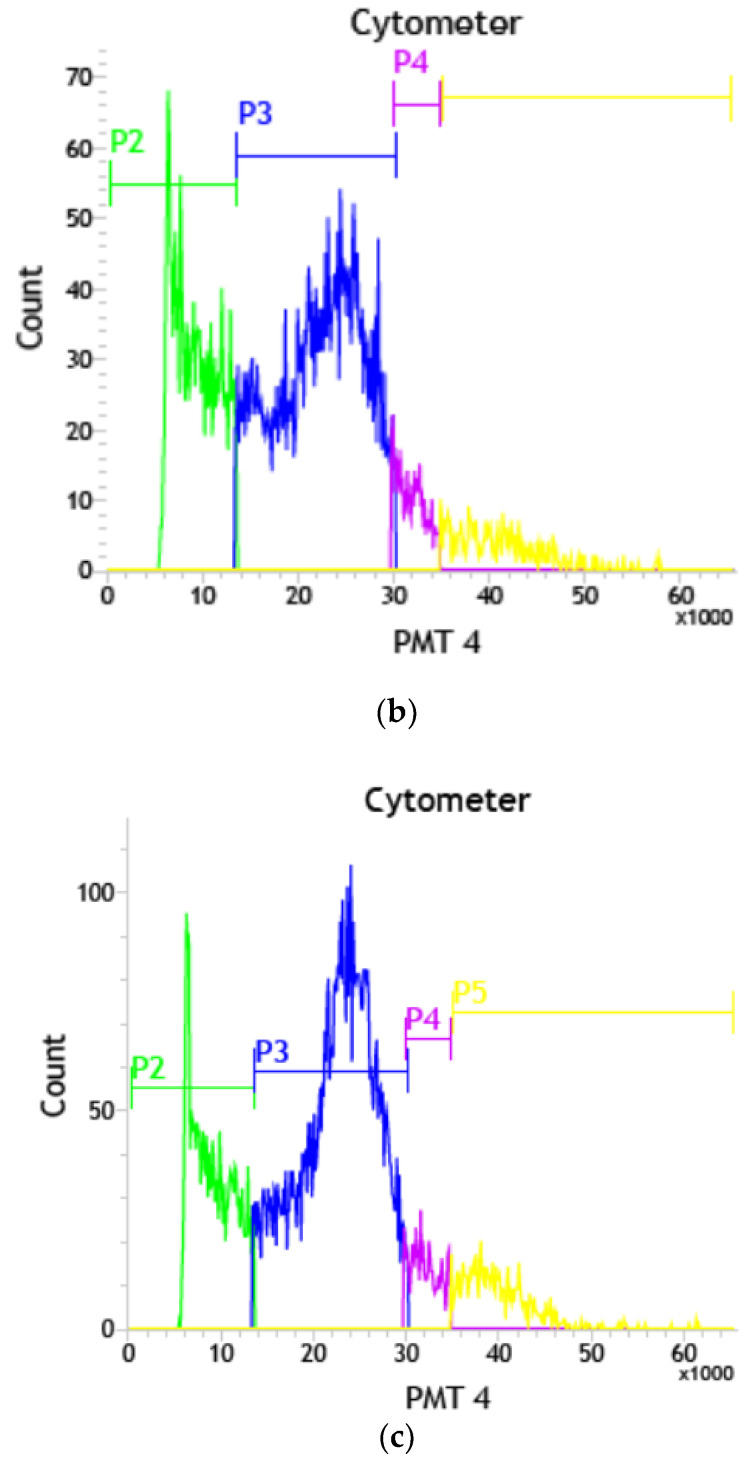
(**a**) Flowcytometric detection of programmed cell death in HT-29 human colon cancer cells—control; (**b**) flowcytometric detection of programmed cell death in HT-29 human colon cancer cells—GO-treated cells; (**c**) flowcytometric detection of programmed cell death in HT-29 human colon cancer cells—rGO-treated cells.

The cell cycle is responsible for cell growth and inhibition [72]. The aberrant regulation of the cell cycle [73,74,75] might be a trigger for apoptosis. HT-29 cells were treated with and without IC_50_ concentration of GO (15.6 µg/mL) and T-rGO (31.2 µg/mL) for 24 h to assess their effects on cell cycle progression. The obtained results indicate that the cell number in the G_1_ phase is found to be proportional to the time of exposure, when the IC_50_ concentration of the GO and T-rGO increases. Thus, HT-29 cells are controlled in the G_1_ and sub-G_0_ stages of the cell cycle by IC_50_ concentrations of GO and T-rGO. Furthermore, with IC_50_ concentrations of GO and T-rGO, HT-29 cells are halted in the sub-G_0_ and G_1_ stages of the cell cycle (Figure 7a–c).

### 3.8. DNA Fragmentation

**Figure 8:**
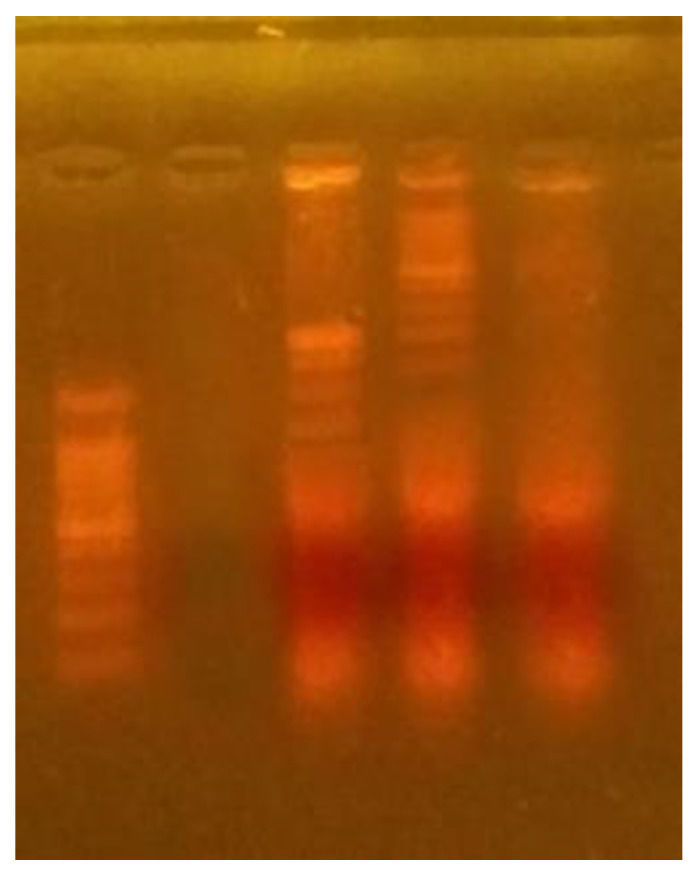
DNA fragmentation of HT-29 human colon cancer cells screened with GO, T-rGO. DNA was separated from treated and untreated HT-29 cells and electrophoresis performed using agarose gel (Lane M, marker), (Lane 1, control), (Lane 2, rGO), (Lane 3, GO).

A DNA fragmentation test that is typical of cell death was used to determine the mechanism of cell death caused by GO/T-rGO. Cells were treated with IC_50_ concentrations of GO for 24 h (15.6 µg/mL) and T-rGO (31.2 µg/mL), and, subsequently, DNA was extracted and examined using agarose gel electrophoresis. Both GO and T-rGO display a typical ladder pattern of intra-nucleosomal fragmentation after treatment. The GO treatment causes considerable fragmentation, but there are no apoptotic cells in the control group (Figure 8). However, T-rGO does not cause any substantial DNA fragmentation in comparison to GO. In 2.0% agarose gels, low-molecular-weight DNA from these cells are resolved. These findings show that GO and T-rGO are effective apoptosis inducers in HT-29 cells.

Carbon materials are reported to cause genotoxicity in glioblastoma cancer cells U87, according to Jaworski et al. [76]. In vivo tests of graphene–gold nanocomposites loaded with daunorubicin causes cell death and limits the development of multidrug-resistant leukemia cells by activating caspase-8 and -3 [77]. The above findings indicate that graphene nanosheets cause DNA breakage in cancer cells when taken simultaneously [78].

### 3.9. Effect of GO on the Morphology of Cell 

To learn more about the cytotoxic effect of GO and T-rGO, and to assess their impact on the morphology of HT-29 cells, an inverted microscope was used. Both GO and T-rGO are found to cause significant time-dependent morphological changes in cells, including cell shape loss, monolayer rupture, and decreased cell adhesion, thereby indicating impaired cell survival.

**Figure 9:**
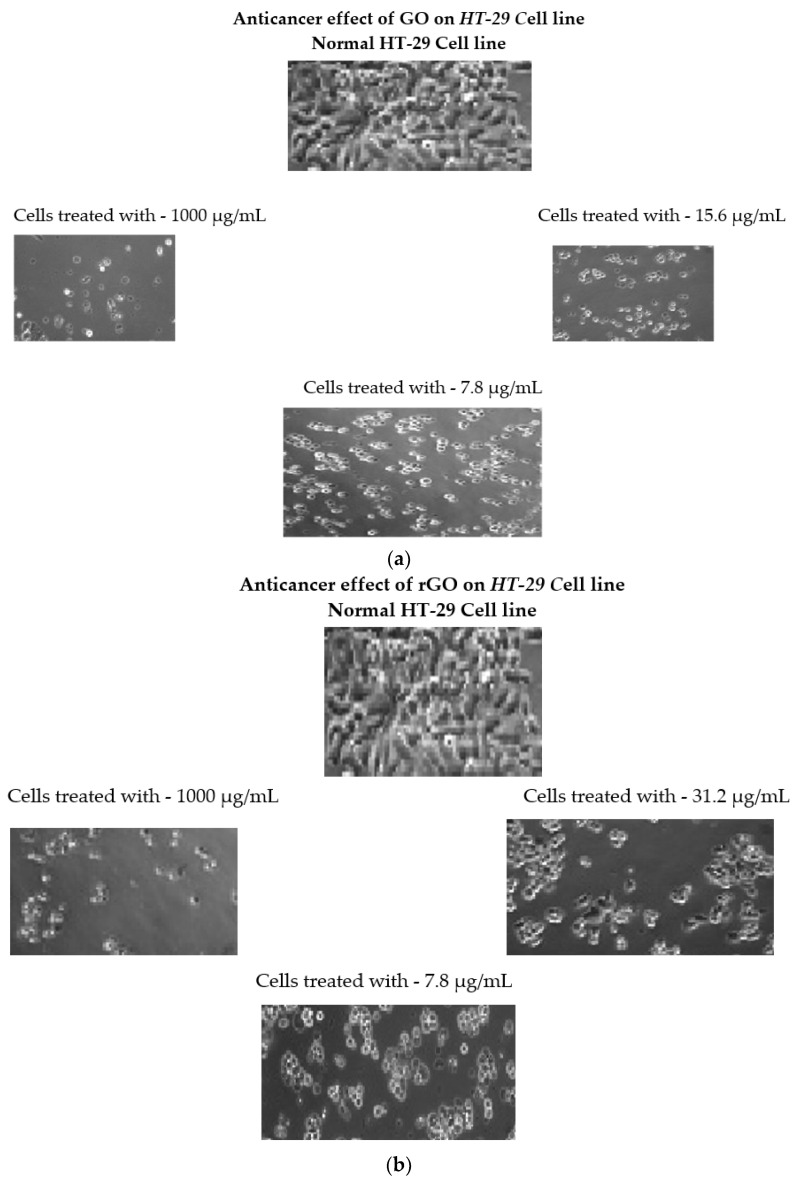
(**a**) Morphology of GO-treated HT-29 human colon cancer cells, (**b**) morphology of T-rGO-treated HT-29 human colon cancer cells. We used 6.3 × 10 magnification to examine the HT-29 cells’ morphology.

Figure 9a,b shows the morphology of HT-29 cell monolayers cultured for 24 h with and without 1000, 15.6, and 7.8 µg/mL GO (IC_50_ concentration) and T-rGO concentrations of 1000, 31.2, and 7.8 µg/mL (IC_50_ concentration).

There is a clear difference between the control and GO-treated cells. The control HT-29 cell monolayers are tiny round bundles of undifferentiated cells with unclear cell margins, whereas GO- and T-rGO-tested cells seem more columnar and epithelial-like, with definite cellular boundaries and longer protrusions than untreated cells. Figure 9a,b shows how GO and T-rGO influence cell structural integrity and substrate adherence. These results are comparable with the results reported by Jian Zhang et al. [79], who find that graphene oxide and reduced graphene oxide impacts rat myocardial cell adhesion.

## 4. Discussion and Conclusions

During the cell viability study, both graphene derivatives demonstrate anticancer activity against colon cancer cells; however GO has a stronger impact than T-rGO. Additionally, it is observed that the cells treated with GO undergo more drastic morphological alterations than cells treated with reduced graphene oxide. It is noteworthy that GO displays significant dose-dependent cytotoxicity by reducing cell viability, cell apoptosis, and DNA laddering of HT-29 human colon cancer cells in comparison to rGO.

The use of thiourea in this study paves the way for the mass manufacture of reduced graphene oxide, a promising nanomaterial that can be employed for cancer nanotherapy, as well as the prevention and treatment of other malignancies.

Lack of selectivity, systemic toxicity, and chemoresistance are reported to be the limitations of conventional chemotherapy approaches to cancer. In this regard, nanomaterials such as graphene could offer more targeted cancer treatment, successfully decreasing undesirable side effects, and offering accurate diagnosis and effective therapy. Nonetheless, limitations remain in the absence of biocompatibility studies, and a lack of bioassays using animal models.

## 5. Perspective and Future Prospects

The contact between T-cells and other antigen-presenting cells can be enhanced using nanocomposites comprised of graphene oxide and reduced graphene oxide. After interaction, the nanoparticles stimulate the immune system by activating T-cells and other antigen-presenting cells. Therefore, the nanocomposites comprising of GO and rGO are crucial for a strong immune response during vaccine development.

## Figures and Tables

**Table 1:** Anticancer effect of GO on HT-29 cell line.

Scheme	Concentration (µg/mL)	Dilution	Absorbance (O.D)	Cell Viability (%)
**1**	1000	Neat	0.042 ± 0.01	4.44
**2**	500	1:1	0.10 ± 0.01	10.13
**3**	250	1:2	0.17 ± 0.01	17.89
**4**	125	1:4	0.29 ± 0.01	24.40
**5**	62.5	1:8	0.35 ± 0.01	35.88
**6**	31.2	1:16	0.43 ± 0.01	44.36
**7**	15.6	1:32	0.50 ± 0.01	51.60
**8**	7.8	1:64	0.53 ± 0.01	55.01
**9**	Cell control	-	0.96 ± 0.01	100

Values of absorbance are presented as mean ± SD.

**Table 2:** Anticancer effect of rGO on HT-29 cell line.

Sample	Concentration (µg/mL)	Dilution	Absorbance (O.D)	Cell Viability (%)
**1**	1000	Neat	0.057 + 0.01	5.89
**2**	500	1:1	0.13 ± 0.01	13.23
**3**	250	1:2	0.26 ± 0.01	26.68
**4**	125	1:4	0.375 ± 0.01	38.78
**5**	62.5	1:8	0.43 ± 0.01	44.26
**6**	31.2	1:16	0.53 ± 0.01	54.80
**7**	15.6	1:32	0.67 ± 0.01	68.97
**8**	7.8	1:64	0.72 ± 0.01	74.14
**9**	Cell control	-	0.97 ± 0.01	100

Values of absorbance are presented as mean ± SD.

## Data Availability

Not applicable.
